# Whole‐genome sequencing and genome‐scale metabolic modeling of *Chromohalobacter canadensis* 85B to explore its salt tolerance and biotechnological use

**DOI:** 10.1002/mbo3.1328

**Published:** 2022-10-26

**Authors:** Blaise Manga Enuh, Belma Nural Yaman, Chaimaa Tarzi, Pınar Aytar Çelik, Mehmet Burçin Mutlu, Claudio Angione

**Affiliations:** ^1^ Biotechnology and Biosafety Department, Graduate and Natural Applied Science Eskişehir Osmangazi University Eskişehir Turkey; ^2^ Department of Biomedical Engineering, Faculty of Engineering and Architecture Eskişehir Osmangazi University Eskişehir Turkey; ^3^ School of Computing, Engineering & Digital Technologies Teesside University Middlesbrough UK; ^4^ Environmental Protection and Control Program Eskişehir Osmangazi University Eskişehir Turkey; ^5^ Department of Biology, Faculty of Science Eskisehir Technical University Eskisehir Turkey; ^6^ Centre for Digital Innovation Teesside University Middlesbrough UK; ^7^ National Horizons Centre Teesside University Darlington UK

**Keywords:** *Chromohalobacter canadensis*, genome‐scale metabolic modeling, halophiles, polyhydroxybutyrates, salt‐tolerant, whole‐genome

## Abstract

Salt tolerant organisms are increasingly being used for the industrial production of high‐value biomolecules due to their better adaptability compared to mesophiles. *Chromohalobacter canadensis* is one of the early halophiles to show promising biotechnology potential, which has not been explored to date. Advanced high throughput technologies such as whole‐genome sequencing allow in‐depth insight into the potential of organisms while at the frontiers of systems biology. At the same time, genome‐scale metabolic models (GEMs) enable phenotype predictions through a mechanistic representation of metabolism. Here, we sequence and analyze the genome of *C. canadensis* 85B, and we use it to reconstruct a GEM. We then analyze the GEM using flux balance analysis and validate it against literature data on *C. canadensis*. We show that *C. canadensis* 85B is a metabolically versatile organism with many features for stress and osmotic adaptation. Pathways to produce ectoine and polyhydroxybutyrates were also predicted. The GEM reveals the ability to grow on several carbon sources in a minimal medium and reproduce osmoadaptation phenotypes. Overall, this study reveals insights from the genome of *C. canadensis* 85B, providing genomic data and a draft GEM that will serve as the first steps towards a better understanding of its metabolism, for novel applications in industrial biotechnology.

## Graphical abstract

The whole genome of *Chromohalobacter canadensis* 85B was sequenced and its genome‐scale metabolic model was reconstructed and validated, enabling a better understanding of its metabolism. We show that *C. canadensis* is a metabolically versatile organism with many features for stress and osmotic adaptation, including pathways for compatible solute and polyhydroxybutyrate (PHB) synthesis. The model reveals the ability to grow on several carbon sources in minimal medium, and produce PHBs and compatible solutes such as ectoine, 5‐hydroxyectoine, and betaine.

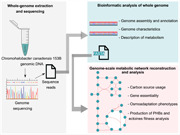

## 1 INTRODUCTION

*Chromohalobacter* is a genus of halophilic bacteria that have evolved methods to survive high salinity environments, with the ability to tolerate up to 12% w/v salt concentration in a minimal medium. They can also have a tolerance in the same environment to other conditions such as pH and temperature, thus widening the applications of their bioproducts (Gedikli et al., 2019). *Chromohalobacter canadensis* is part of the Halomonadaceae within the phylum Bacteria. The clade is made up of *Chromohalobacter marismortui, Chromohalobacter canadensis, Chromohalobacter israelensis, Chromohalobacter salexigens, Chromohalobacter beijerinckii, Chromohalobacter japonicus, Chromohalobacter nigrandensis, Chromohalobacter salarius*, and *Chromohalobacter saracensis* (Arahal & Ventosa, 2006).

To survive high salinity and low water activity in their environment, halophilic bacteria use salt‐in and low salt‐in strategies as well as nutrient storage strategies. The salt‐in strategy involves the accumulation of inorganic salts such as KCl to balance the osmotic difference with the environment. The low‐salt‐in strategy involves the accumulation of organic solutes also called compatible solutes, which allow enzymes and other cellular processes to function properly. Organic compounds that have been identified as compatible solutes include polyols, sugars, amino acids, betaines, ectoines, N‐acetylated diamino acids, and N‐derivatized carboxamides of glutamine (Gunde‐Cimerman et al., 2018). Surprisingly, these adaptations have also evolved to make their metabolism more efficient in high salinity and less efficient in low salinity (Pastor et al., 2013). They have also adapted to using a wide variety of simple carbon compounds as sole carbon sources and having high energy‐rich polymer reserves. One such compound is polyhydroxybutyrate (PHB), a type of polyhydroxyalkanoate (PHA). The PHAs are candidate biodegradable bioplastics to replace currently used plastics that are a source of environmental pollution. These unique adaptation mechanisms offer a rich source of exploitable bacterial bioresource.

The physiology of halophiles and the range of bioproducts they can synthesize make them suitable for use as industrial cell factories. Halophilic organisms’ resilience to extreme conditions translates to reduced chances of contamination in industrial bioreactors. Their enzymes, (Prakash et al., 2009) exopolysaccharides and osmoprotectants also have several industrial applications contributing to making them highly attractive as industrial cell factories. *C. canadensis* has been shown to produce PHBs, ectoines, amylases, and other high‐value industrial products (Prakash et al., 2009; Radchenkova et al., 2018; Wang et al., 2020). Their potential for bioremediation has also been reported (Erdogmus et al., 2015). Recent research also shows a promising potential in the production of levan, which is a high‐value polymer in cosmetics and also safe for consumption (Çakmak et al., 2020). Within the *Chromohalobacter* clade, however, the genomics and in silico analysis of *C. salexigens* (Ates et al., 2011; Copeland et al., 2011) has been better studied compared to *C. canadensis* and other members. Despite the reported potential applications of *C. canadensis*, there is little information on the potential of *C. canadensis* from a genomic insight, which can be exploited for future metabolic engineering and systems biology research.

Advances in technology and computational biology tools are driving current research in biotechnology (Becker & Wittmann, 2018). High throughput technologies such as whole‐genome sequencing allow in‐depth insight into the potential of organisms. Using whole genomes, detailed metabolic processes of organisms and their phenotypic characteristics under various external conditions are increasingly revealed with genome‐scale metabolic network models (GEM) (Fang et al., 2020; Gu et al., 2019). These models are stoichiometry‐based mathematical descriptions that permit the modeling of biochemical metabolic pathways in living systems.

Recently, more sophisticated semi‐automated tools for the reconstruction of GEMs have been developed that build genome‐scale models from annotated genomes though need minimal manual curation and validation before use (Gu et al., 2019; Machado et al., 2018). Flux balance analysis (FBA) and its variations can be subsequently used to investigate the metabolic phenotypes for various environmental and genetic perturbations, predicting flux rates of all known biochemical reactions in a variety of conditions (Orth et al., 2010). Genomic insights into halophilic metabolism have revealed different synthetic pathways that affect the PHA type produced. Hence, state‐of‐the‐art systems biology tools such as GEMs can facilitate the contextualization of metabolism for specific strains that can be used for production optimization studies (Mitra et al., 2020). The GEMs are at the frontier of systems biology and, when combined with data mining or machine learning methods, are increasingly driving novel biotechnological discoveries. For example, omics data and GEMs are being exploited by novel machine and deep learning algorithms to tackle a variety of research questions in biotechnology, ranging from maximization of yield to characterization of growth across conditions (Ben Guebila & Thiele, 2019; Culley et al., 2020; Enuh & Aytar Çelik, 2022; Kavvas et al., 2020; Vijayakumar et al., 2020; Zampieri et al., 2019). By providing a platform exploitable by researchers from a wide range of disciplines, GEMs enable a better understanding of metabolism, driving novel applications and discoveries in industrial biotechnology (Fang et al., 2020).

Here, we sought to obtain insight from the whole genome of *C. canadensis* 85B about its metabolism by using high throughput sequencing, annotation, and analyses of its genes. Using a semiautomated pipeline, we then built and curated a GEM from the annotated genome. We standardized and validated the model against experimental data from the literature. Our model can provide an in silico platform for *C. canadensis* that can be used for future studies, using genome‐scale models for applications in biotechnology.

## 2 METHODS

### 2.1 Bacteria strains

Bacteria samples were obtained from stored slant cultures that were isolated from another study (Çakmak et al., 2020) and inoculated on a nutrient agar medium for 24 h to revive. From the nutrient agar medium, an inoculum was obtained and transferred to a minimal salt medium composed of NaCl (96 g), MgCl_2_.6H_2_O (12 g), MgSO_4_.7H_2_O (14 g), KCl (2.8 g), NaBr (0.32 g), NaHCO_3_ (0.008 g), CaCl_2_.2H_2_O (2 g), yeast extract (1 g), Peptone (5 g), and glucose (20 g) as carbon source. The culture was incubated for 3 days at 35°C and 150 rpm in 250 mL Erlenmeyer flasks for polymer production (Dyall‐Smith, 2015).

### 2.2 Genomic DNA extraction

From the bacterial cultures, 2 mL of bacterial suspension was obtained for genomic DNA extraction. Genomic DNA was extracted using the PureLink Microbiome DNA purification kit (Invitrogen) according to the manufacturer's instructions. Upon extraction of the pure DNA, an electrophoresis gel was prepared to confirm the presence of a single band corresponding to the whole bacterial genome. A 5 µL of the sample was run on 1% agarose gel for 30 min at 100 v. Gels were stained with ethidium bromide (10 mg mL^−1^) and visualized on a gel documentation system (BIO‐RAD).

### 2.3 Genome sequencing and annotation

The genomic DNA samples were sent for genome sequencing to BM laboratories and sequenced with the Illumina NGS sequencing platform. After sequencing, quality analysis was done with FASTQc v0.11.9 to obtain raw reads quality and trimming was done with default settings. The sequence reads were assembled and ordered with the Unicycler pipeline (Wick et al., 2017) in PATRIC (https://www.patricbrc.org/) using the auto assembly strategy with default parameters (Wattam et al., 2017, 2018). Unicycler first produces an Illumina assembly graph, then uses long reads to build bridges and anchors to determine the positions of the contigs. This allowed resolving all repeats in the genome, resulting in a complete genome assembly. The replicons were then circularized and rotated to begin at a consistent starting gene.

The genome was annotated using the RAST tool kit v3.6.9 (RASTtk) (Brettin et al., 2015) annotation pipeline provided through the RAST annotation web service (https://rast.nmpdr.org) and PATRIC (Wattam et al., 2018). Further annotation with an orthology‐based search to complement the homology annotations from RAST was done with Evolutionary Genealogy of Genes: Non‐supervised Orthologous Groups (EggNOG) (Huerta‐Cepas et al., 2019) to assign functional annotation to the detected orthologous groups and to facilitate the interpretation results from RAST homology predictions. The KAAS (Moriya et al., 2007) annotation server with BLAST and BBH (bidirectional best hit) was used for pathway reconstruction. When needed, metabolic pathways were further inferred from the KEGG database (http://www.genome.jp/kegg/) (Kanehisa & Goto, 2000) and BioCyc (Karp et al., 2019).

Gene features of essential biosystems were also further confirmed manually using BLASTp (https://blast.ncbi.nlm.nih.gov/Blast.cgi). Predicted complementary DNA sequences were blasted in the NCBI nonredundant database as well as Swiss‐Prot and UniProt, (Boutet et al., 2007), and the information was combined to obtain the characteristics of proteins. Genomic features and characteristics were displayed with the circular genome viewer tool server (CGView) (Stothard et al., 2019) for generating genomic maps for microorganisms using the annotated genome from the RAST server.

### 2.4 Phylogenetic analysis

The 16 S ribosomal subunit sequences were obtained from the annotated genome and a sequence blast was done in the NCBI database. The first 35 hits were selected and used to generate the phylogenetic tree in Molecular Evolutionary Genetics Analysis MEGA X (Kumar et al., 2018).

### 2.5 Genome‐scale modeling

#### 2.5.1 Draft metabolic model reconstruction

CarveMe v1.4.1 (Machado et al., 2018) was used with default pipeline arguments to curate a draft reconstruction from the genome of *C. canadensis* 85B. So, CarveMe is an automated pipeline that uses a top‐down method to build both single‐species and community models rapidly and with high scalability. The pipeline leverages the BIGG database for metabolite and reaction information. These models perform closely to manually curated models in terms of reproducing experimental phenotypes such as gene essentiality and substrate utilization. The genome file with annotations was retrieved in the FASTA format from the RAST server and passed into the CarveMe pipeline with $ carve ‐‐dna genome.fna arguments in the command line for reconstruction.

#### 2.5.2 Model benchmarking

The metabolic model testing suite, MEMOTE v0.11.1 (Lieven et al., 2020) in its command‐line version was used to benchmark the model against standardized principles of model descriptions and to obtain a report that can be used for further model curation. The results of the standard tests and annotations helped direct further curation of the model for consistency, metabolic gaps, assigning metabolite charges, and reaction bounds. The MEMOTE reports were iteratively generated after manual curation steps to ensure the highest possible score (Lieven et al., 2020).

#### 2.5.3 Addition of annotations

To extend the annotations in the model, ModelPolisher v2.0.1 was used (Römer et al., 2016). ModelPolisher compares the model's entity IDs to the BiGG model database and retrieves relevant metadata compliant with SBO terms (Schellenberger et al., 2010). All relevant information and data about the matching instance are integrated as annotations into the initial draft reconstruction for each related entry in the BiGG database.

#### 2.5.4 Manual curation and gap analysis

After the initial draft was curated and annotated, manual refinement steps followed. All manual steps were conducted by refining the model in COBRApy v0.22.1. (Ebrahim et al., 2013). Literature evidence related to *C. canadensis* (Arahal & Ventosa, 2006; Radchenkova et al., 2018) was used to verify the reactions in the model as well as to add reactions, metabolites, or genes that were missing due to annotation errors. Annotation information from RAST and EggNOG served as sources to trace the presence of genes and gene ontologies respectively. For reactions that were added to the model, appropriate scores based on the information obtained from the literature were also noted. Blocked metabolites were identified using COBRApy (Ebrahim et al., 2013). The identifiers were used to search the KEGG (Kanehisa & Goto, 2000) and Biocyc (Karp et al., 2019) databases that served as a reference to curate missing reactions and fill metabolic gaps. When present, the reactions were verified for mass and charge balance and corrected, when necessary, before inclusion. The output model was tested for SBML compliance with the COBRApy library in Python 3.8.

#### 2.5.5 Minimal medium

Metabolite essentialities in the medium were carefully verified by limiting each metabolite's availability and subsequently optimizing the model. If the in silico simulations revealed no growth after limiting the metabolite's availability, the metabolite's essentiality was considered confirmed. Finally, the list of media components that were essential was used to make up the minimal medium for the model.

#### 2.5.6 Model validation and analysis

Using the minimal medium obtained from simulations, the in silico growth capabilities of *C. canadensis* 85B on different carbon sources were examined. All available sugar exchange fluxes were extracted from the model and sorted into monosaccharides, disaccharides, oligosaccharides, and trisaccharides. For the exchange reactions of the carbon source under investigation, the lower bound was set to −10 mmol gDW^−1^ h^−1^. Each carbon source was tested individually by only enabling the tested carbon source's exchange reaction and by optimizing the model for growth using FBA (Orth et al., 2010). Simulations with a flux value of zero were considered as an inability for the model to grow on the carbon source used. Further investigations of reaction fluxes in optimal states were done with Flux Variability Analysis (FVA), setting the biomass flux to its maximal FBA value, therefore with a fraction of the optimum value of 1.0 (Mahadevan & Schilling, 2003), and the fitness in producing bioproducts was investigated with a phenotypic phase plane analysis using CAMEO (Cardoso et al., 2018) in python 3.8.

#### 2.5.7 Visualization

To facilitate model curation and analyzing pathways, Escher was used for visualizing the fluxes in the model's metabolic pathways. Escher enables the building of metabolic pathways using reactions, metabolites, and genes by contextualizing them in the organism's metabolism (King et al., 2015). The Escher Python package v1.7.1 (King et al., 2015) was also used to draw customized metabolic maps of *C. canadensis* 85B in Jupyter notebooks as it is compliant with COBRApy. Graphs for carbon source predictions were plotted with ggplot2 (Wickham, 2009) in R studio version 4.1.1 (RStudio Team, 2015).

## 3 RESULTS AND DISCUSSIONS

### 3.1 Genomic properties

The genome was assembled after sequencing and according to basic statistics, the genome length was estimated to be 3,718,005 bp, there were 34 contigs with protein‐encoding genes (PEGs) and an average G + C content of 60.90%. The N50 length, which is defined as the shortest sequence length at 50% of the genome, was 186,789 bp. The L50 count, which is defined as the smallest number of contigs whose length sum produces N50, was 5 (Table 1). Very few studies have reported the genome sequence of bacteria in the *Chromohalobacter* genus. A comparison of genome properties for *Chromohalobacter* genomes reported in the literature is shown in Table 2. Considering that the genus contains nine species, it shows that there is still a lot of research to be done to understand the physiology and potential of *Chromohalobacter*.

**Table 1:** Summary features for *Chromohalobacter canadensis* 85B whole genome

Characteristic	Value
Size	3,718,005
GC content	60.90
N50	186,789
L50	5
Number of contigs (with PEGs)	34
Number of subsystems	315
Number of coding sequences	3478
Number of RNAs	70

**Table 2:** Comparison of the genomic features of *Chromohalobacter canadensis* 85B of this study with other *Chromohalobacter* species.

Species	Genome length (bp)	Protein coding sequences	GC content (%)	Reference
*C. canadensis 85B*	3,718,005	3478	60.9	This study
*C. marismortui DSM 6770*	3,553,220	3226	61.7	(RefSeq: NZ_SOBR00000000.1),
*C. salexigens type strain (1H11* ^*T*^ *)*	3,696,649	3319	63.9	Copeland et al. (2011)
*C. salexigens ANJ207*	3,664,372	3344	63.71	Srivastava et al. (2019)
*Chromohalobacter sp. SMB17*	3,775,557	3486	60.5	Olsson et al. (2017)
*C. israelensis DSM 6768^T^*	3,660,991	3361	63.74	Zhou et al. (2015)

*Note*: Only completed assemblies were considered with a taxonomy check confirmed. A lower GC content but a higher number of predicted coding sequences were observed with *C. canadensis* 85B.

**Figure 1:**
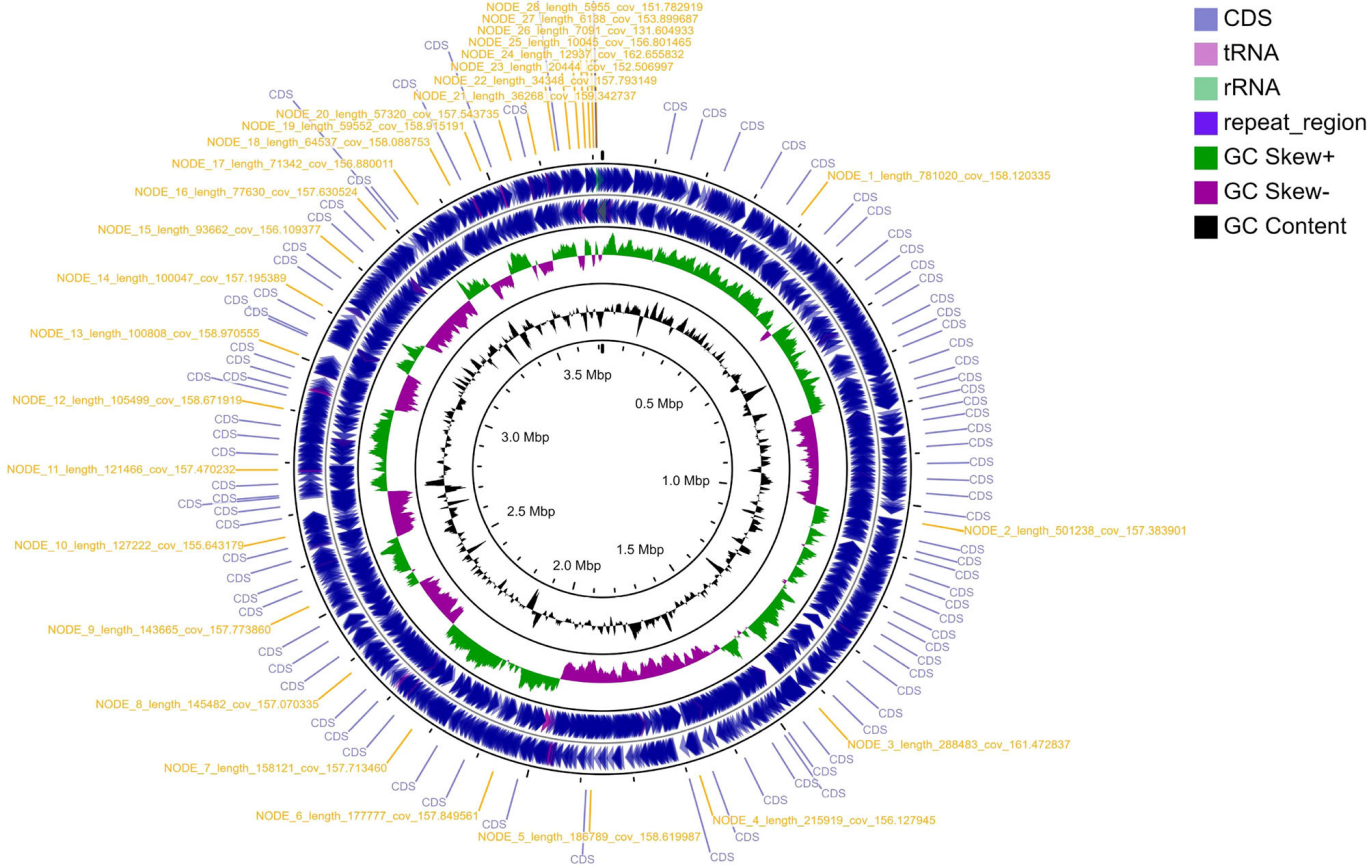
Circular map showing the distribution of genes in *Chromohalobacter canadensis* 85B genome. Ordered from the outer ring to the inner rings are contigs with their labels, forward and reverse strands of CDS, RNA genes, GC skew, and GC content.

A circular graphical display of the distribution of the genome annotations is provided (Figure 1). This includes, from outer to inner rings, the contigs with contig code labels, CDS on the forward and the reverse strand also labeled as CDS; RNA genes are embedded within the forward and reverse strand rings; the GC skew and GC content are also shown in the same order.

### 3.2 Phylogenetic analysis

**Figure 2:**
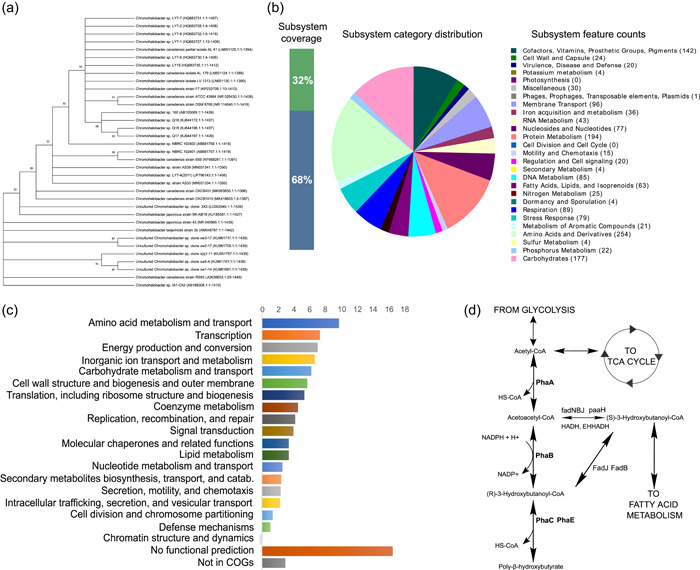
(a) Phylogenetic tree showing the relationship between *Chromohalobacter canadensis* 85B and other microorganisms. The accession numbers and length of sequences used are shown in brackets (b) Subsystems in the *C. canadensis* 85B genome. (c) Number of genes associated with general COG functional categories. (d) Polyhydroxybutyrate (PHB) synthesis pathway prediction according to KEGG. Intermediates from both glycolysis and fatty acid metabolism. (S)‐3‐Hydroxybutanoyl‐CoA is an important intermediate as it links the PHB synthesis pathway and fatty acid metabolism. fadN, fadB, fadJ and fadB, and fadJ, are fatty acid degradation enzymes, 3‐hydroxybutyryl‐CoA dehydrogenase [EC:1.1.1.157] (paaH), 3‐hydroxyacyl‐CoA dehydrogenase [EC:1.1.1.35] (HADH), EHHADH.

The 16 S ribosomal subunit sequences were obtained from the annotated genome, and a sequence blast was performed in the NCBI database. The evolutionary history was inferred using the Neighbor‐Joining method (Saitou & Nei, 1987). The bootstrap consensus tree inferred from 1000 replicates was taken to represent the evolutionary history of the taxa analyzed (Felsenstein, 1985). Branches corresponding to partitions reproduced in less than 50% of bootstrap replicates were collapsed. The percentage of replicate trees in which the associated taxa clustered together in the bootstrap test (1000 replicates) are shown next to the branches (Felsenstein, 1985). The evolutionary distances were computed using the Maximum Composite Likelihood method (Tamura et al., 2004) and are in the units of the number of base substitutions per site. This analysis involved 35 nucleotide sequences. All ambiguous positions were removed for each sequence pair (pairwise deletion option). There were a total of 1449 positions in the final data set. Evolutionary analyses were conducted in MEGA X (Kumar et al., 2018). Similar to the above‐mentioned close relatives, an identity of 99.79% was reported for *C. canadensis* strain DSM 6769^T^ and *C. canadensis* strain ATCC 43984^T^ 99.79% followed by *C. japonicus* 99.38%. This agrees with the classification of the *Chromohalobacter* genus that had previously been established based on the closer sequence similarity to other *Chromohalobacter* members (Arahal et al., 2001). Relationships with other strains are shown in the phylogenetic tree (Figure 2a).

### 3.3 Overview of subsystems and orthologous cluster genes

A subsystem is a set of proteins that together implement a specific biological process or structural complex. Thirty‐two percent (1080) of annotated proteins were included in the subsystems analysis according to the RAST pipeline. An overview of the subsystems for this genome as produced by the annotation pipeline is provided in Figure 2b. The amino acids and derivates form the highest proportion of subsystem annotations followed by carbohydrate metabolism, protein metabolism, cofactors, and membrane transport. Proteins play an important role in the adaptation of halophiles to high salinity. This suggests that *C. canadensis* 85B possesses the machinery to meet its adaptation needs in a saline environment. The same is also observed for the membrane transport systems. Osmolite balance is fundamental for halophiles therefore robust membrane transport systems ensure that the integrity of the cell is maintained with changing conditions.

An analysis of orthologous genes shows amino acid metabolism and transport and transcription containing the highest number of orthologous genes (Figure 2c). When compared with results by Copeland et al. (2011) on *C. salexigens* the first seven groups seem to be the most abundant despite the subtle differences in the relative abundance of orthologous genes between both species. This further emphasizes the importance of these systems in this group of microorganisms.

### 3.4 Carbohydrate metabolism

There were 177 carbohydrate metabolism genes in *C. canadensis* 85B and nine subsystems representing biosynthesis and degradation pathways. Predictions show genes for the metabolization of various carbohydrate substrates such as sugar alcohols, C‐1 compounds, sugar acids, monosaccharides, polysaccharides, and fermentation. Enzymes able to metabolize the following substrates were predicted: glucose, starch, sucrose, fructose, mannose, xylose, glycerol, and galactose. The presence of many different pathways for carbohydrate metabolism has significant implications for the adaptation of halophiles.

In *C. salexigens*, glucose metabolism occurs exclusively through the Entner–Doudoroff pathway while fructose metabolism occurs through the Entner–Doudoroff and Embden–Meyerhof–Parnas pathways. Fructose metabolism seems to give more metabolic flexibility in response to energy and biosynthetic demands. The Entner–Doudoroff pathway, on the other hand, is inefficient for growth when salinity is low, as a result of metabolite overflow. However, in high salinity, there is a high metabolic burden on this pathway due to the use of NADPH and ATP for the synthesis of compatible solutes. This allows the organism to use other pathways to meet other metabolic requirements (Pastor et al., 2019).

Despite the closeness of both species, the Entner–Duodoroff pathway was not predicted in *C. canadensis* 85B, therefore other adaptation mechanisms may apply. Other studies show that halophilic bacteria may prefer to metabolize glucose only after other substrate sources are depleted (Oren & Mana, 2003). Experimental studies with *C. canadensis* are needed to derive conclusions as this will be helpful for organism‐specific approaches. The broad range of usable carbohydrate substrates is a biotechnology advantage through the growth on a wide variety of possible cheap substrates which can help reduce production costs (Güngörmedi et al., 2014).

### 3.5 Fatty acid metabolism

The fatty acid composition of salt‐tolerant organisms is influenced by salt concentrations. This is observed through decreased saturation of fatty acids at suboptimal concentrations. Therefore by varying the ratio of saturated to unsaturated fatty acids adaptation to salt stress can be achieved (Mutnuri et al., 2005). This shows the important role of fatty acid metabolism in the adaptation of organisms living in high salinity. In the *C. canadensis* 85B genome, there were five subsystems and 63 genes predicted to be involved in fatty acid metabolism. Pathways for fatty acid, phospholipids triacylglycerols, and isoprenoid metabolism were predicted. The KEGG annotations show both fatty acid biosynthesis and fatty acid degradation pathways. Fatty acid degradation occurs through beta‐oxidation which also has Acetoacetyl‐CoA and (S) ‐3‐Hydroxybutanoyl‐CoA intermediates that link it to the PHB synthesis pathway.

### 3.6 Stress response, defense, and virulence

The main types of stress response systems identified were osmotic stress, heat/cold shock, stress, resistance to antibiotics and toxic compounds, and the *Hfl* operon; details are presented in Table 3 below. In bacteria, glutathione plays an important role in protecting the cell from the effects of low pH, chlorine chemicals, and oxidative and osmotic stressors, in addition to maintaining the appropriate oxidation state of protein thiols. Furthermore, by directly modifying proteins via glutathionylation, glutathione has emerged as a posttranslational regulator of protein function under oxidative stress (Masip et al., 2006). Iron homeostasis regulators have previously been shown to play a role in the complicated circuit that governs halophilic bacteria's response to osmotic stress in *C. salexigens* (Masip et al., 2006).

**Table 3:** Predicted stress response and defense systems

Subclass	Subsystem name	Gene count	Role count
Resistance to antibiotics and toxic compounds	Antibiotic targets in DNA processing	4	4
	Resistance to Triclosan	1	1
	Fusaric acid resistance cluster	6	3
	Beta‐lactamases Ambler class C	1	1
	Antibiotic targets in metabolic pathways	5	4
	Polymyxin resistance, lipid A modifications with phosphoethanolamine	2	2
	Antibiotic targets in transcription	3	3
	Antibiotic targets in protein synthesis	8	8
	Mupirocin resistance	1	1
	Copper homeostasis: Copper tolerance	2	2
	Antibiotic targets in cell wall biosynthesis	3	3
	Resistance to Daptomycin	4	3
	Fusidic acid resistance	2	2
	Cadmium resistance	1	1
	Resistance to chromium compounds	1	1
Stress Response	Repair of iron centers	4	3
	Glutathione: Redox cycle	3	3
	Glutathione: Non‐redox reactions	8	5
	Cluster containing glutathione synthetase	4	4
	Glutathione: Biosynthesis and gamma‐glutamyl cycle	4	3
	Protection from reactive oxygen species	7	7
	Stress proteins YciF, YciE	2	2
	Universal stress protein family	1	1
Stress Response: Heat/cold shock	Heat shock *dnaK* gene cluster extended	17	16
	Cold shock proteins of CSP family	4	1
Stress Response: Osmotic stress	Choline uptake and conversion to betaine clusters	34	21
	Ectoine, hydroxyectoine uptake and catabolism	8	7
	Ectoine synthesis	7	7
	Osmoregulation	1	1
	Glycine betaine synthesis from choline	4	4
	Hyperosmotic potassium uptake	3	2
Other	Hfl operon	5	5

### 3.7 Polyhydroxyalkanoates

In some organisms, the genes for PHA are frequently located on the same operon but in *C. canadensis* the PHA genes were located on different loci in the genome. The genes identified were *PhaA, PhaB, PhaC*, and *PhaR* (Table 4). The *PhaA* gene was predicted in two locations on the genome while others were found in one location only. Note, PHA synthase (*PhaC*) is the key enzyme in the PHB synthesis pathway, catalyzing the polymerization of hydroxyalkanoate subunits (Figure 2d). Note, PHA synthase influences the type of monomer, the composition, and the weight of the PHA produced (Zheng et al., 2020). Four classes of PHA synthases have been identified based on their primary sequence, the composition of subunits, and their substrate specificities. Class I PHA synthases are homodimers, class II is made of PhaC1 and PhaC2 subunits, class III is made of PhaC and PhaE, and class IV PhaC and PhaR. Classes I, III, and IV produce short‐chain length monomers made of three to five carbon lengths while class II synthases produce six to 14 carbon chain lengths (Chek et al., 2017). Up to 14 different pathways for PHB synthesis have been described so far leading to the production of homopolymers, random copolymers, block copolymers, and graft polymers (Meng et al., 2014).

**Table 4:** Predicted polyhydroxybutyrate biosynthesis genes and their genomic characteristics

Function	Ontology	Aliases	Start	Strand	Length	Contig
3‐ketoacyl‐CoA thiolase (EC 2.3.1.16) @ Acetyl‐CoA acetyltransferase (EC 2.3.1.9)	SSO:000000312‐ 3‐ketoacyl‐CoA thiolase (EC 2.3.1.16)	PhbA	17,888	+	1182	NODE_2_length_501238_cov_157.383901
	SSO:000000702‐ Acetyl‐CoA acetyltransferase (EC 2.3.1.9)					
3‐ketoacyl‐CoA thiolase (EC 2.3.1.16)	SSO:000000312‐ 3‐ketoacyl‐CoA thiolase (EC 2.3.1.16)	PhbA	25,498	‐	1179	NODE_20_length_57320_cov_157.543735
Acetoacetyl‐CoA reductase (EC 1.1.1.36)	SSO:000000675‐ Acetoacetyl‐CoA reductase (EC 1.1.1.36)	PhbB	12,237	+	747	NODE_1_length_781020_cov_158.120335
Polyhydroxyalkanoic acid synthase		PhaC	730,278	+	1857	NODE_1_length_781020_cov_158.120335
Polyhydroxyalkanoate synthesis repressor PhaR		PhaR	33,110	+	459	NODE_6_length_177777_cov_157.849561

The protein sequence of the PHA synthase gene was blasted in NCBI to assess the type of PHA synthase enzyme. Blast results returned 99.51% similarity with *C. japonicus*, 99.35% *C. salexigens*, and 98.38% *C. canadensis*. A further search by blast in the Uniprot database first hit 99.5% similarity with Class I poly(R)‐hydroxyalkanoic acid synthase (*C. japonicus*). Only one hit was obtained each in the Gene3D, InterPro, Pfam, SUPFAM, and TIGRFAMs, all corresponding to PHA synthase class I. The class I subfamily PHA synthases can polymerize hydroxyacyl‐CoAs with three to five carbons in the hydroxyacyl into PHA esters in this case most likely PHB. These can be accumulated up to 90% of the cell's dry weight. The PhaR genes play a posttranscriptional role and help prevent protease degradation or act directly or indirectly to activate PHA synthase (McCool & Cannon, 2001). Note, PhaR is found to be a DNA‐binding homotetramer that is also capable of binding short‐chain hydroxyalkanoic acids and PHA granules. Thus, PhaR may regulate the expression of itself, the phasins that coat granules, and enzymes that direct carbon flux into polymers stored in granules (Maehara et al., 2002). Further research to determine the specific function of PhaR in PHB synthesis in *C. canadensis* is required.

**Figure 3:**
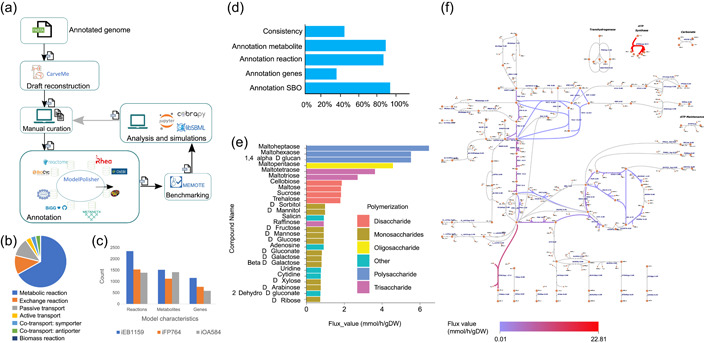
(a) Model development process from reconstruction from the annotated genome to refinements and analysis. (b) Distribution of metabolic reaction types in the model. (c) Comparison of iEB1159 model and two models of *C. salexigens* iOA584 and iFP764. (d) MEMOTE test for model benchmarking. (e) Carbon sources that were shown to produce growth in minimal media and their corresponding fluxes. (f) Escher map of Glucose metabolism showing the flow of metabolites and the distribution of flux in the central carbon metabolism pathway. The colors represent different flux ranges as shown in the legend.

According to KEGG annotations, fadNBJ, paaH, HADH, EHHADH, fadJ, and fadB enzymes are from the fatty acid metabolism pathways. As shown in Figure 2d, (S)‐3‐Hydroxybutanoyl‐CoA can be either isomerized to (R)‐3‐Hydroxybutanoyl‐CoA or converted to Acetocetyl‐CoA which are both intermediates in the PHB synthesis pathway. This suggests that fatty acid metabolism and PHB synthesis in *C. canadensis* 85B are closely related. Hence, under the right conditions, fatty acid metabolism can deviate toward the production of PHBs. Similar observations have been made with *Halomonas sp*. SF2003 (Thomas et al., 2019).

### 3.8 Genome‐scale modeling and analysis

#### 3.8.1 General model features

After reconstructing the draft, the model development followed an iterative path (Figure 3a). The initial draft model contained 1522 metabolites, 2347 reactions, and 1159 genes within three compartments: the cytosol, periplasm, and extracellular space. The model was named iEB1159 according to the model naming convention, with *i* representing in silico, EB the initials of the name of the model curator, and 1159 the number of genes in the model. There are 1830 annotated reactions in the model. The distribution of reaction types according to their SBO categories is shown in Figure 3b. A comparison of general model features with other previously reported *Chromohalobacter* models iFP764 (Piubeli et al., 2018) and iOA584 (Ates et al., 2011) is reported in Figure 3c, showing that iEB1159 has a larger number of reactions, genes, and metabolites.

#### 3.8.2 Model benchmarking

The initial model results in MEMOTE returned a score of 37%, with the lowest scores due to poor annotations. After model curation and the addition of annotations, a MEMOTE score of 70% was achieved. Considering that this is the first genome‐scale model of *C. canadensis* 85B and the lack of data to fill gaps, we believe that this is a promising score, showing the model has a good foundation for research improvement (Figure 3d).

#### 3.8.3 Addition of annotations

Models by CarveMe produce annotations in the Notes area of the model. However, this is not detected by MEMOTE during benchmarking. Annotations for metabolites, SBO terms, and genes included in the model permitted a high score with MEMOTE. ModelPolisher permitted the inclusion of annotations in the right fields that can be identified by MEMOTE. Annotation databases that were queried include BiGG (Schellenberger et al., 2010), BioCyc (Karp et al., 2019), CHEBI (Degtyarenko et al., 2008), HMDB (Wishart et al., 2007), Inchikey (Heller et al., 2015), Lipidmaps (Liebisch et al., 2020), KEGG (Kanehisa and Goto, 2000), Reactome (Fabregat et al., 2018), SEED (Seaver et al., 2020), MetaNetX (Moretti et al., 2021), and EC‐code, RHEA (Alcántara et al., 2012).

Further SBO terms annotations were done manually using the libSBML package in Python according to the SBO conventions (http://www.ebi.ac.uk/sbo/main/). The annotations are as follows: passive transport (SBO:0000658), active transport (SBO:0000657), cotransport:symport (SBO:0000659), cotransport:antiport (SBO:0000660) other transport reactions (SBO:0000655), general metabolic reactions (SBO:0000176), exchange reactions (SBO:0000627), biomass reactions (SBO:0000629), genes (SBO:0000243), and species (SBO:0000247) (Figure 3b).

#### 3.8.4 Gap analysis

There were 37 blocked metabolites identified in the model. Further investigation of metabolites using the BIGG database showed that the blocked reactions were mostly exchange reactions, cofactors, and prosthetic groups. Escher maps enabled visualization of metabolic pathways that served to identify incomplete pathways for gap filling (Figure 3f). Due to the lack of data on *C. canadensis* in the major databases, most of the pathway gaps could not be investigated in‐depth. These were allowed and considered as knowledge gaps that will be filled with growing research. There was however high metabolite connectivity as reported by MEMOTE with a score of 100%. The output model was further tested for SBML compliance with the COBRApy (Ebrahim et al., 2013) library in Python, and all errors were corrected. The final model contains all SBML fields as required.

#### 3.8.5 Minimal medium

The minimal medium for the model was obtained by iteratively checking for growth in the model in limiting conditions. During simulations, glucose was maintained as the sole carbon source while the entrance of simple salts and ions was varied. The secretion of other carbon‐containing compounds was monitored to ensure that only CO_2_ was produced in the final medium. The final number of essential metabolites termed the minimal media are provided in Table A2. (Table A1)

#### 3.8.6 Validation of carbon source usage

Microorganisms in the Halomonadaceae family are metabolically diverse. Within individual species, the ability to support growth on a carbon source can vary between studies (Arahal & Ventosa, 2006). Genome‐scale models provide a systems approach to understanding the interplay between carbon sources, metabolic pathway dynamics, and the biosynthesis of important metabolites (Ates, 2015). Model predictions are important in guiding experiments requiring labeling or for the production of specific bioproducts. With this in mind, FBA simulations on a wide range of carbon sources were carried out with iEB1159 to assess its ability to represent carbon use phenotypes and reproduce experimental results.

In silico predictions were done by considering biomass as an objective function, with glucose as the sole carbon source on the minimal medium previously obtained. Growth on other carbon sources was simulated with FBA by using each carbon source in separate simulations as the sole source of carbon with an uptake value of 10 mmol/gDW/h. Overall, the model showed growth on 27 carbon sources (Figure 3e), with varying flux rates. The high biomass yield of greater than 2 g/mmol for some carbon sources could be attributed to the need to determine the precise uptake rate for such substrates, as 10 mmol/gDW/h was obtained from other organisms. It was also observed that the polymerization of the carbon source influenced the growth rate, with the growth rate increasing as the level of polymerization increased. To provide a context for the results obtained, the predictions were compared with experimental data previously reported (Arahal & Ventosa, 2006; Radchenkova et al., 2018). The model did not grow on lactose, citrate, and esculin as shown in previous studies (Arahal & Ventosa, 2006; Radchenkova et al., 2018), despite the presence of citrate and both l‐lactose and d‐lactose transport reactions. This suggests an important gap in knowledge that requires further attention considering that lactose is a favorable substrate in the production of exopolysaccharides (Radchenkova et al., 2018). Thus, iEB1159 also predicted growth in several carbon sources not previously studied (Table 5).

**Table 5:** Comparison of *Chromohalobacter canadensis* growth on various carbon sources reported in the literature and in silico predictions of iEB1159

Compound name	Experimental	Insilico	Reference
d‐Glucose	+	+	Arahal & ventosa (2006)
Maltose	−	+	Arahal & ventosa (2006)
Maltotriose	No data	+	no report
d‐Arabinose	+	+	Arahal & ventosa (2006)
Cellobiose	+	+	Arahal & ventosa (2006)
d‐Fructose	+	+	Arahal & ventosa (2006)
d‐Galactose	No data	+	no report
Beta d‐Galactose	No data	+	no report
d‐Gluconate	No data	+	no report
Maltoheptaose	No data	+	no report
Maltohexaose	No data	+	no report
Maltopentaose	No data	+	no report
Maltotetraose	No data	+	no report
d‐Mannose	No data	+	no report
d‐Mannitol	No data	+	no report
Raffinose	No data	+	no report
d‐Ribose	−	+	Arahal & ventosa (2006)
d‐Sorbitol	−	+	Arahal & ventosa (2006)
Sucrose	+	+	Arahal & ventosa (2006)
Trehalose	No data	+	no report
d‐Xylose	+	+	Arahal & ventosa (2006)
Esculin	+	not in model	Arahal & ventosa (2006)
l‐Rhamnose	not determined	−	Arahal & ventosa (2006)
Starch	Varies	not in model	Arahal & ventosa (2006)
Citrate	+	−	Arahal & ventosa (2006)
Fumarate	not determined	−	Arahal & ventosa (2006)
Adonitol	not determined	not in model	Arahal & ventosa (2006)
l‐Lysine	not determined	−	Arahal & ventosa (2006)
Lactose	+	−	Radchenkova et al. (2018)
1,4‐alpha‐d‐glucan	No data	+	no report
2‐Dehydro‐Dgluconate	No data	+	no report
Adenosine	No data	+	no report
Cytidine	No data	+	no report
Uridine	No data	+	no report
Salicin	No data	+	no report

The model did not grow in anaerobic conditions, confirming its strictly aerobic phenotype (Ventosa & Haba, 2020). When oxygen was limited, no growth was produced by the model even in the presence of a potential electron acceptor such as Fe^3+^. So, *C. salexigens* iOA584 was reported to grow anaerobically on nitrate (Ates et al., 2011); for iEB1159, no growth was observed using nitrate in anaerobic conditions despite the presence of transport and other metabolic reactions. Such differences are the basis for hypotheses for research to either improve the model knowledge base or better understand microbial cellular behaviors.

#### 3.8.7 Osmoadaptation phenotypes

Salt tolerance is a hallmark phenotype of halophilic organisms with several mechanisms happening simultaneously for survival. The uptake and synthesis of compatible solutes constitute an important adaptation strategy for *Chromohalobacter* (Arahal & Ventosa, 2006; Piubeli et al., 2018). According to the genome annotation, *C. canadensis* 85B should be able to oxidize choline to betaine and synthesize ectoine de novo via the use of *EctA*, *EctB*, and *EctC* genes. In addition, these pathways also seem to be evolutionarily conserved in halophilic ectoine producers (Arahal & Ventosa, 2006; Piubeli et al., 2018).

Ectoine and 5‐hydroxyectoine were included in the biomass reaction and their respective amounts were calculated from the amounts in the *C. salexigens* model by Piubeli et al. (2018) in relation to NaCl molarity. This provides a useful approximation because both species are close and share similar salinity adaptation features. Demand reactions were also included to simulate the production of intracellular ectoine. Our FBA simulations at optimal growth showed states with flux in the direction of ectoine synthesis and the production of small amounts of glycine betaine when choline was added to the medium. According to Thiele and Palsson (2010); demand functions can be added for compounds that the organism is known to produce, and for which its production is dependent on environmental conditions. This enables the reactions to become active like in their favorable environment (Thiele & Palsson, 2010). This can become useful for our model when simulating osmoadaptation phenotypes. Simulations show that ectoine synthesis is inversely related to growth. Besides, the synthesis of ectoine is highly regulated and requires specific conditions. This can be correlated with the fact that ectoine synthesis is energy‐intensive, also reported with the iFP764 model (Piubeli et al., 2018).

It is worth noting that when product biosynthesis rates are predicted, FBA simulations do not take into account the impact of gene regulation as they only predict optimal solutions. Hence, when validating simulations in vivo, culture conditions that provide optimal responses need to be determined to match in silico FBA predictions. In such cases, in principle, FBA predictions suggest optimal product biosynthesis rates after regulatory genes have been knocked out in cases when these genes are known (O'Brien et al., 2015). To further improve the quality and scope of predictions related to osmoadaptation, experiments towards determining the precise biomass compositions in different salinities, and integrating other omics data into the model are encouraged. This will be important in understanding osmoadaptation in *C. canadensis* and halophiles in general.

#### 3.8.8 Gene essentiality

The analysis of the essential genes in iEB1159 was done by doing single‐gene knockout simulations and then optimizing the model for growth. When growth was not predicted, the knocked‐out gene and its associated reactions were considered essential. In total, 60 essential genes were predicted (Table A2). Most essential genes were those related to the metabolism of amino acids and nucleotides, ectoine synthesis as well as the transportation of ions. Specifically, our model predicted the Cl^‐^ channel (voltage‐gated), and zinc/iron permease which have been reported to be associated with adaptations to high salt environments by sensing salt stress and regulating intracellular ion homeostasis respectively (Ding et al., 2019; He et al., 2020). Noteworthy is that the mechanism through which voltage‐gated Cl^‐^ channel contributes to salt tolerance is not yet clearly understood. Our model could provide a platform to integrate transcriptomics data to further investigate these mechanisms using a systems biology perspective (Occhipinti et al., 2021).

#### 3.8.9 Model fitness to produce PHBs and ectoine

Halophilic bacteria are well known for their ability to produce PHBs and ectoine which alongside other physiological mechanisms enable survival in conditions of high salt concentrations. The PHBs are energy‐rich compounds accumulated under nutrient‐limiting conditions, while ectoines are compatible solutes that help maintain a growth‐supporting osmotic balance for the cell. Both are high‐value products with several uses in the biotechnology industry (Prakash et al., 2009; Radchenkova et al., 2018; Wang et al., 2020).

**Figure A1:**
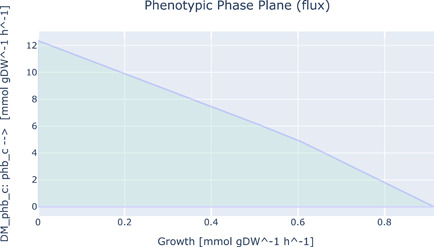
Phenotypic phase plane for Polyhydroxybutyrate production.

**Figure A2:**
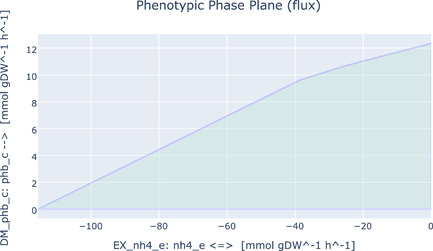
Phase plane analysis of Polyhydroxybutyrate production with varying concentrations of nitrogen source.

**Figure A3:**
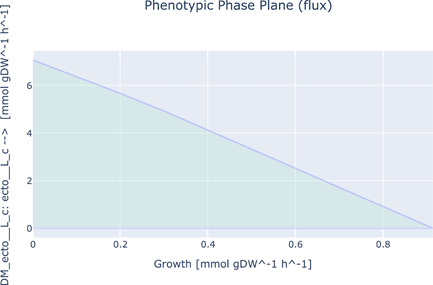
Phenotypic phase plane for ectoine production with varying biomass.

**Figure A4:**
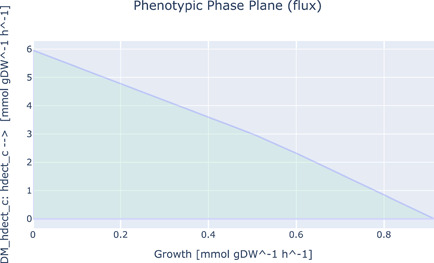
Phenotypic phase plane for 5‐ hydroxyectoine production with varying biomass.

To investigate the ability of iEB1159 to produce PHBs and ectoines, First, the model was simulated with FBA for optimal growth, and the flux of the reactions producing both products was recorded. Secondly, FVA was done to investigate the existence of other potential optimal states. Thirdly, the objective function was changed to the demand reaction in the respective pathways producing both products and simulated to observe their highest possible production rate. Finally, a phenotypic phase plane analysis to investigate the fitness of the model to produce these metabolites at optimal conditions was performed and plotted (Figures A1, A2, A3, A4).

For PHB synthesis, FVA simulations showed a minimum and maximum flux of 0.0 mmol/gDW/h and 12.35 mmol/gDW/h respectively. The fitness of iEB1159 to produce PHBs showed that its production is inversely proportional to the growth rate and that up to 12.35 mmol/gDW/h of PHBs could be produced with the lowest possible growth rate (Figure A1). The phase plane analysis with PHB synthesis and nitrogen source uptake (NH_4_^+^) showed a decrease in PHB production with increasing nitrogen uptake rates, although with a steeper slope after uptake rates of about 39 mmol/gDW/h (Figure A2). This suggests that in vivo, if *C. canadensis* reaches optimal growth, decreasing the uptake rate of NH_4_^+^ to trigger secondary metabolism will result in a fairly proportional increase in PHB production. These predictions are in agreement with laboratory and industrial PHB production fermentation schemes (Koller, 2018; McAdam et al., 2020). Therefore, iEB1159 shows the potential to accurately predict the production dynamics of PHBs.

The fitness of iEB1159 to produce ectoine showed that its production is inversely proportional to the growth rate and that up to 7.05 mmol/gDW/h of ectoine could be produced when the growth rate is lowered (Figure A3). A similar trend was also observed for 5‐hydroxyectoine (Figure A4). This could be explained by the fact that the synthesis of ectoine draws significant amounts of intermediates from the TCA cycle, which reduces their availability for other growth‐associated processes, thereby affecting the growth rate (Piubeli et al., 2018).

## 4 CONCLUSIONS

Halophilic bacteria have enormous biotechnological potential, and there is growing interest in using them as alternative resilient cell factories and sources of high‐value bioproducts. Their use towards this end requires an understanding of their genetics and physiology to better design strategies that exploit their potential. In this study, the complete genome sequence of *C. canadensis* 85B was analyzed and a draft genome‐scale model was built to provide a base for future systems biology research. We hope that this model will provide the first computational tool to improve our understanding of its metabolism and drive novel biotechnology discoveries.

Generally, the genome of *C. canadensis* 85B is comparable to the genome of other Chromohalobacter, and genes for adaptation and production of high‐value products were predicted. The analysis of metabolic subsystems showed that carbohydrate metabolism was the second‐largest important pathway, indicating the importance for the organism to obtain and transform a wide variety of carbon sources in diverse ways to obtain energy. This is also supported by the pathway diversity predicted for metabolizing different carbon compounds and producing energy. For environment‐specific adaptation, according to the COG functional categories, the transport of inorganic ions and metabolism contained up to 233 genes. Salt and ion balance are very important for adaptation to saline environments as previously reported by other studies (Oren, 1999; Ventosa et al., 1998). The stress response system was dominated by glutathione and ectoine. Studies on other halophiles show the use of similar systems to mitigate stress and ectoine for osmotic stress (Cai et al., 2011; Pastor et al., 2012; Schwibbert et al., 2011). *C. canadensis* 85B grows at high salinity in which compatible solutes such as ectoine are necessary for adaptation. Of interest is also the production of polyhydroxyalkanoate biopolymers as high‐energy stores.

We here built a GEM of the metabolism of *C. canadensis* 85B. First, we generated a draft reconstruction which was further curated, annotated, and used for simulations in an iterative fashion. Finally, we validated the model with literature data. Our model provides a platform for multi‐omic data integration and potential combination with machine learning and deep learning approaches. Compared to other organisms like *E. coli* or *S. cerevisiae*, there is a limited pool of specific experimental data on *C. canadensis*, indicating that there are still many knowledge gaps and opportunities for exploration, especially for use in condition‐specific modeling and optimization (Czajka et al., 2021; Vijayakumar & Angione, 2021; Zhang et al., 2020).

The validated draft metabolic network model reconstructed in this study can be updated in line with all GEMs, and can be further improved with context‐specific modeling approaches, for instance in presence of condition‐specific omics data. Nevertheless, we note that GEMs remain powerful tools even when the knowledge base is not yet complete. For instance, the model built here correctly predicts the growth on different carbon sources in minimal media, and the production of ectoines, betaine, and PHBs. We hope that researchers from a wide range of disciplines will be able to use the model to further understand its metabolism, driving novel hypotheses on its use in industrial biotechnology.

## AUTHOR CONTRIBUTIONS

**Blaise Manga Enuh**: Conceptualization (equal), Formal analysis (equal), Funding acquisition (equal), Visualization (equal), Writing – review & editing (equal). **Belma Nural Yaman**: Conceptualization (equal), Funding acquisition (equal), Writing – review & editing (equal). **Chaimaa Tarzi**: Formal analysis (equal), Visualization (equal), Writing – review & editing (equal). **Pınar Aytar Çelik**: Conceptualization (equal), Funding acquisition (equal), Supervision (equal), Writing – review & editing (equal). **Mehmet Mutlu**: Supervision (equal), Writing – review & editing (equal). **Claudio Angione**: Conceptualization (equal), Funding acquisition (equal), Supervision (equal), Visualization (equal), Writing – review & editing (equal).

## CONFLICT OF INTEREST

None declared.

## ETHICS STATEMENT

None required.

## Data Availability

The data that support the findings of this study are available in the Appendix. The whole genome shotgun project is available in DDBJ/ENA/GenBank under the accession JAJQJH000000000: https://www.ncbi.nlm.nih.gov/nuccore/JAJQJH000000000. The genome‐scale metabolic model is available in the BioModels database with the identifier MODEL2204110001: https://www.ebi.ac.uk/biomodels/MODEL2204110001 and on GitHub: https://github.com/Angione-Lab/GEM-Chromohalobacter-canadensis-85B.

## 1

**Table A1:** Minimal media

Metabolite identifier	Metabolite name
ca2_e	Calcium
cl_e	Chloride
cobalt2_e	Cobalt
cu2_e	Copper
fe2_e	Ferrous Iron
glc__D_e	d‐Glucose
k_e	Potassium
mg2_e	Magnesium
mn2_e	Manganese
nh4_e	Ammonium
o2_e	Oxygen
pi_e	Phosphate
so4_e	Sulfate
zn2_e	Zinc

**Table A2:** Essential genes predicted by iEB1159

Gene ID	Growth	Growth status	Gene product
{‘fig_141389_9_peg_2976'}	0	optimal	Phosphomethylpyrimidine synthase ThiC (EC 4.1.99.17)
{‘fig_141389_9_peg_2742'}	0	optimal	3'(2'),5'‐bisphosphate nucleotidase (EC 3.1.3.7)
{‘fig_141389_9_peg_1758'}	0	optimal	3‐methyl‐2‐oxobutanoate hydroxymethyltransferase (EC 2.1.2.11)
{‘fig_141389_9_peg_230'}	0	optimal	Dihydrofolate synthase (EC 6.3.2.12) @ Folylpolyglutamate synthase (EC 6.3.2.17)
{‘fig_141389_9_peg_1773'}	0	optimal	Phosphoglucosamine mutase (EC 5.4.2.10)
{‘fig_141389_9_peg_1804'}	0	optimal	Argininosuccinate lyase (EC 4.3.2.1)
{‘fig_141389_9_peg_861'}	0	optimal	Threonine synthase (EC 4.2.3.1)
{‘fig_141389_9_peg_3064'}	0	optimal	3‐dehydroquinate dehydratase II (EC 4.2.1.10)
{‘fig_141389_9_peg_884'}	0	optimal	Deoxyuridine 5'‐triphosphate nucleotidohydrolase (EC 3.6.1.23)
{‘fig_141389_9_peg_1913'}	0	optimal	Dihydroorotase (EC 3.5.2.3)
{‘fig_141389_9_peg_2658'}	0	optimal	Undecaprenyl diphosphate synthase (EC 2.5.1.31)
{‘fig_141389_9_peg_1532'}	0	optimal	3‐isopropylmalate dehydrogenase (EC 1.1.1.85)
{‘fig_141389_9_peg_2718'}	0	optimal	Phosphoribosylformimino‐5‐aminoimidazole carboxamide ribotide isomerase (EC 5.3.1.16)
{‘fig_141389_9_peg_3119'}	0	optimal	UDP‐N‐acetylmuramoyl‐l‐alanine‐‐d‐glutamate ligase (EC 6.3.2.9)
{‘fig_141389_9_peg_2809'}	0	optimal	Thymidylate kinase (EC 2.7.4.9)
{‘fig_141389_9_peg_1215'}	0	optimal	N‐acetyl‐gamma‐glutamyl‐phosphate reductase (EC 1.2.1.38)
{‘fig_141389_9_peg_860'}	0	optimal	Homoserine dehydrogenase (EC 1.1.1.3)
{‘fig_141389_9_peg_1423'}	0	optimal	Serine acetyltransferase (EC 2.3.1.30)
{‘fig_141389_9_peg_3232'}	0	optimal	S‐adenosylmethionine synthetase (EC 2.5.1.6)
{‘fig_141389_9_peg_2716'}	0	optimal	Imidazoleglycerol‐phosphate dehydratase (EC 4.2.1.19)
{‘fig_141389_9_peg_1530'}	0	optimal	3‐isopropylmalate dehydratase large subunit (EC 4.2.1.33)
{‘fig_141389_9_peg_2630'}	0	optimal	Phosphoribosyl‐ATP pyrophosphatase (EC 3.6.1.31)
{‘fig_141389_9_peg_2668'}	0	optimal	N‐succinyl‐L,l‐diaminopimelate desuccinylase (EC 3.5.1.18)
{‘fig_141389_9_peg_1982'}	0	optimal	Cl^‐^ channel, voltage gated
{‘fig_141389_9_peg_415'}	0	optimal	Pantothenate kinase type III, CoaX‐like (EC 2.7.1.33)
{‘fig_141389_9_peg_3186'}	0	optimal	Argininosuccinate synthase (EC 6.3.4.5)
{‘fig_141389_9_peg_882'}	0	optimal	N‐acetylglutamate kinase (EC 2.7.2.8)
{‘fig_141389_9_peg_226'}	0	optimal	Phosphoribosylanthranilate isomerase (EC 5.3.1.24)
{‘fig_141389_9_peg_2779'}	0	optimal	Cysteine synthase B (EC 2.5.1.47)
{‘fig_141389_9_peg_948'}	0	optimal	Branched‐chain amino acid aminotransferase (EC 2.6.1.42)
{‘fig_141389_9_peg_683'}	0	optimal	Indole‐3‐glycerol phosphate synthase (EC 4.1.1.48)
{‘fig_141389_9_peg_3097'}	0	optimal	UDP‐N‐acetylglucosamine 1‐carboxyvinyltransferase (EC 2.5.1.7)
{‘fig_141389_9_peg_3118'}	0	optimal	Phospho‐N‐acetylmuramoyl‐pentapeptide‐transferase (EC 2.7.8.13)
{‘fig_141389_9_peg_2514'}	0	optimal	Tol‐Pal system‐associated acyl‐CoA thioesterase
{‘fig_141389_9_peg_310'}	0	optimal	Erythronate‐4‐phosphate dehydrogenase (EC 1.1.1.290)
{‘fig_141389_9_peg_1942'}	0	optimal	zinc/iron permease
{‘fig_141389_9_peg_2717'}	0	optimal	Imidazole glycerol phosphate synthase amidotransferase subunit HisH
{‘fig_141389_9_peg_2824'}	0	optimal	UDP‐N‐acetylenolpyruvoylglucosamine reductase (EC 1.3.1.98)
{‘fig_141389_9_peg_340'}	0	optimal	FMN adenylyltransferase (EC 2.7.7.2)/Riboflavin kinase (EC 2.7.1.26)
{‘fig_141389_9_peg_3117'}	0	optimal	UDP‐N‐acetylmuramoyl‐tripeptide‐‐d‐alanyl‐d‐alanine ligase (EC 6.3.2.10)
{‘fig_141389_9_peg_3156'}	0	optimal	Orotidine 5'‐phosphate decarboxylase (EC 4.1.1.23)
{‘fig_141389_9_peg_1963'}	0	optimal	N‐acetylglucosamine‐1‐phosphate uridyltransferase (EC 2.7.7.23)/Glucosamine‐1‐phosphate N‐acetyltransferase (EC 2.3.1.157)
{‘fig_141389_9_peg_306'}	0	optimal	Dihydroorotate dehydrogenase (quinone) (EC 1.3.5.2)
{‘fig_141389_9_peg_885'}	0	optimal	Phosphopantothenoylcysteine decarboxylase (EC 4.1.1.36)/Phosphopantothenoylcysteine synthetase (EC 6.3.2.5)
{‘fig_141389_9_peg_1707'}	0	optimal	GTP cyclohydrolase I (EC 3.5.4.16) type 1
{‘fig_141389_9_peg_274'}	0	optimal	NAD kinase (EC 2.7.1.23)
{‘fig_141389_9_peg_3145'}	0	optimal	Phosphoserine aminotransferase (EC 2.6.1.52)
{‘fig_141389_9_peg_2879'}	0	optimal	Flavin prenyltransferase UbiX
{‘fig_141389_9_peg_3146'}	0	optimal	Chorismate mutase I (EC 5.4.99.5)/Prephenate dehydratase (EC 4.2.1.51)
{‘spontaneous'}	0	optimal	#N/A
{‘fig_141389_9_peg_3220'}	0	optimal	5,10‐methylenetetrahydrofolate reductase (EC 1.5.1.20)
{‘fig_141389_9_peg_3099'}	0	optimal	Histidinol dehydrogenase (EC 1.1.1.23)
{‘fig_141389_9_peg_1899'}	0	optimal	Orotate phosphoribosyltransferase (EC 2.4.2.10)
{‘fig_141389_9_peg_3123'}	0	optimal	d‐alanine‐‐ ligase (EC 6.3.2.4)
{‘fig_141389_9_peg_1807'}	0	optimal	Diaminopimelate epimerase (EC 5.1.1.7)
{‘fig_141389_9_peg_1531'}	0	optimal	3‐isopropylmalate dehydratase small subunit (EC 4.2.1.33)
